# Development of an RPE-Based Prediction Model for Trunk Muscle Activation During Water Inertia Load Exercise: A Pilot EMG Study

**DOI:** 10.3390/jfmk11010089

**Published:** 2026-02-21

**Authors:** Shuho Kang, Ilbong Park

**Affiliations:** Department of Sports Rehabilitation, Busan University of Foreign Studies, Busan 46234, Republic of Korea; 20236204@bufs.ac.kr

**Keywords:** water inertia load, surface electromyography, rating of perceived exertion, unstable resistance exercise: trunk muscle activation, prediction model

## Abstract

**Background:** Water inertia load training using equipment such as water vests provides unstable resistance that enhances trunk muscle activation. However, practical methods for prescribing exercise intensity without expensive electromyography (EMG) equipment remain limited. This pilot study aimed to develop prediction models for estimating trunk muscle activation using rating of perceived exertion (RPE) during water inertia load exercises. **Methods:** Seventeen healthy adults (20.45 ± 2.02 years) performed lateral trunk flexion exercises wearing a water vest at five progressive loads (8–16 kg in 2 kg increments). Surface EMG was recorded from four trunk muscles (rectus abdominis, external oblique, internal oblique, erector spinae) and normalized to maximal voluntary isometric contraction (%MVIC). Rating of perceived exertion (RPE) was assessed using the Borg CR-10 scale. Load-dependent changes in muscle activation were examined using repeated-measures ANOVA, and relationships between RPE and EMG were analyzed using regression and linear mixed-effects models. **Results:** All trunk muscles showed significant increases in activation with increasing load (all *p* < 0.001, ηp^2^ = 0.381). RPE demonstrated significant positive correlations with all abdominal muscles (r = 0.37–0.46, *p* < 0.001). Simple regression analyses indicated predictive accuracy (R^2^ = 0.267), representing a 29% increase compared with the strongest individual muscle model. Linear mixed-effects modeling confirmed RPE as a significant predictor after accounting for inter-individual variability. **Conclusions:** This pilot study provides preliminary evidence that RPE can be used to estimate trunk muscle activation during water inertia load exercise. The proposed composite activation index enhances prescription when EMG measurement is not feasible.

## 1. Introduction

Instability resistance training, unlike traditional exercises performed on stable surfaces, is known to be effective in enhancing proprioception and neuromuscular control by intentionally introducing variability into the external environment [1]. According to systematic reviews, trunk muscle activation during core stabilization exercises varies depending on exercise type, with rectus abdominis, external oblique, and erector spinae showing different activation patterns [2]. Notably, unstable conditions significantly increase trunk muscle activation compared to stable conditions [3]. Equipment utilizing water inertia load has gained attention as a form of instability training. Water inertia load equipment, such as water bags and water vests, possesses unique inertia characteristics in which the internal liquid moves in a delayed manner opposite to the direction of movement, thereby substantially increasing neuromuscular responses required for contraction timing control, dynamic balance maintenance, and trunk stabilization [4,5]. Importantly, water inertia load differs qualitatively from surface-based instability devices such as Swiss balls, BOSU balls, and wobble boards, which provide predictable instability in relatively fixed planes and shift postural control toward hip-dominant strategies due to reduced reliability of ankle proprioceptive signals [6]. Moreover, moderately unstable surfaces have been shown to fail in enhancing muscle activation in resistance-trained individuals [7]. In contrast, water inertia generates rapid, irregular, and continuous external perturbations on stable ground surfaces that cannot be anticipated or pre-programmed, requiring ongoing compensatory trunk muscle activation with each movement cycle [6]. This unpredictable, multidirectional nature of water inertia may explain why it elicits greater trunk stabilization demands than conventional instability equipment. Several studies have reported that water inertia load equipment can induce higher trunk muscle activation than fixed weights of equivalent absolute mass [8,9,10]. Recently, Kang and Park [6] reported that water inertia load training significantly improved balance ability in older women. These findings suggest that water inertia provides dynamic and unpredictable characteristics beyond simple resistance, playing an important role in enhancing trunk stability.

Surface electromyography (sEMG) is a representative bio signal technology that can quantitatively assess muscle activation during exercise and has been widely utilized in research related to exercise intensity and load adjustment. However, sEMG has limitations in practical training setting due to high equipment costs and complex attachment and analysis procedures. In contrast, rating of perceived exertion (RPE) is a tool that can rapidly assess exercise intensity based on subjective judgment and has shown close associations with various physiological and neuromuscular indicators [11,12]. In particular, significant correlations between sEMG and RPE have been reported (r = 0.30–0.80), and RPE sensitively reflects muscle fatigue and increased muscle activation not only in resistance exercise but also in continuous exercise [13,14]. Helms et al. [15] also emphasized that RPE is a reliable indicator for monitoring muscle load intensity. Furthermore, Zhao et al. [16] recently reported a significant correlation (r = 0.573) between RPE and muscle fatigue indicators during back squat exercise, supporting the validity of RPE for predicting muscle activation. However, studies providing objective criteria for trunk muscle activation according to load changes during water inertia load training remain very limited. Previous studies have indicated that increased water inertia enhances muscle activation [4,9], but they have not provided elements necessary for practical exercise prescription, such as muscle activation changes by load, the quantitative relationship with RPE, or muscle activation prediction models. In particular, despite the unstable characteristics of water bags potentially leading to individually preferred muscle activation strategies, no research has considered this aspect. Therefore, field practitioners have no choice but to rely on subjective judgment when selecting loads for water inertia load equipment, which remains an important limitation in terms of standardizing and ensuring safety in exercise intensity prescription. From a neurophysiological perspective, RPE is believed to reflect the magnitude of central motor command (corollary discharge) sent to working muscles, rather than peripheral afferent feedback alone [15]. During unstable resistance exercise, the central nervous system must generate additional motor commands to coordinate reactive stabilization against unpredictable perturbations, which may be reflected in elevated RPE. This theoretical link between central motor drive and perceived exertion provides the rationale for using RPE as a surrogate indicator of trunk muscle activation during water inertia load exercise. Accordingly, this study aimed to (1) examine changes in trunk muscle activation with increasing water inertia load (8–16 kg), (2) analyze the quantitative relationship between RPE and muscle activation, (3) develop a composite trunk activation index controlling for individual differences in muscle activation strategies, and (4) establish an RPE-based prediction model that enables practitioners to estimate trunk muscle activation using only subjective RPE without EMG equipment, thereby providing preliminary evidence for evidence-based exercise intensity prescription during water inertia load training.

## 2. Materials and Methods

### 2.1. Participants

A priori power analysis was conducted using G*Power 3.1.9.7 to determine the required sample size. Based on a repeated-measures ANOVA design (5 load conditions) with a medium effect size (f = 0.25), α = 0.05, and power = 0.80, a minimum of 15 participants was required. Post hoc power analysis using G*Power 3.1.9.7, based on the smallest observed effect size (ES: ηp^2^ = 0.381, f = 0.785), confirmed that statistical power exceeded 0.99 for the final sample of 17 participants, indicating that the study remained adequately powered despite three participant withdrawals. Twenty participants were initially recruited, considering potential dropouts; however, three participants withdrew due to scheduling conflicts, resulting in a final sample of 17 participants (11 males and 6 females; mean age 20.2 ± 1.7 years) for data analysis. Detailed participant characteristics are presented in Table 1. Inclusion criteria were: (1) Age 19–35 years. (2) Regular physical activity experience, including resistance training, aerobic exercise, or sports participation (at least twice per week for more than 6 months). None of the participants had prior experience with water inertia training or other structured instability resistance exercise programs, ensuring that the observed muscle activation patterns were not influenced by previous adaptation to unstable loads. (3) Voluntary consent to participate. (4) Ability to understand and accurately respond to RPE scale instructions in Korean. Exclusion criteria were: (1) musculoskeletal or neurophysiological disorders, (2) injuries within the past 6 months, (3) medication use that could affect study performance, and (4) skin conditions that could affect EMG measurement. All participants provided written informed consent, and this study was approved by the Institutional Review Board (IRB No. P01-202601-01-003) and was prospectively registered at the Clinical Research information Service (CRIS: KCT0011452).

### 2.2. Experimental Design and Water Vest Exercises Protocol

**Figure 1:**
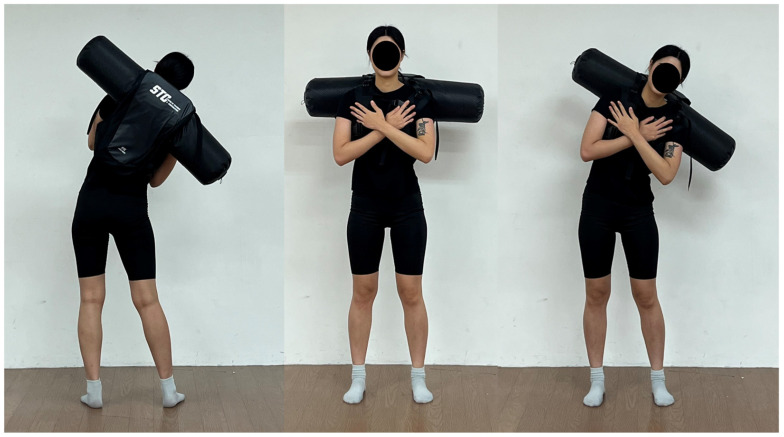
Lateral flexion exercise with water vest.

This study employed a within-subjects repeated-measures design with a crossover approach. Participants performed lateral trunk flexion exercises while wearing a water vest at five progressive loads (8,10,12,14, and 16 kg) (Figure 1). Prior to the main experiment, all participants wore the water vest with a light load (3–5 kg) to familiarize themselves with the sensation of water inertia and were screened for any lower-back pain or discomfort before proceeding. The starting load of 8 kg was selected as the minimum weight at which participants could clearly perceive water inertia during lateral flexion. The 2 kg increment was adopted because initial testing with 1 kg increments revealed that participants were unable to distinguish differences between adjacent load conditions. The upper limit of 16 kg was determined as the maximum load that all participants could safely complete while maintaining proper movement quality. Surface EMG was recorded from four trunk muscles (rectus abdominis, external obliques, internal obliques, and erector spinae), and RPE was assessed using the Borg CR-10 scale after each load condition.

**Figure 2:**
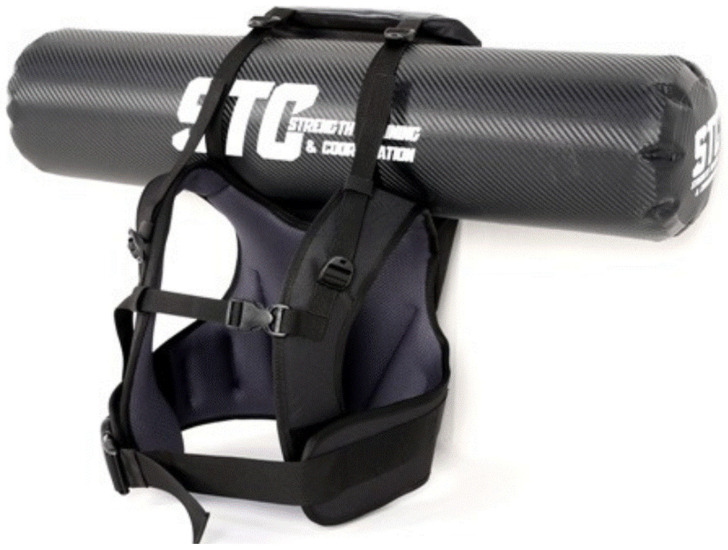
Water vest.

A commercially available water vest (AQUA VEST, SMARTFIT, Busan, Korea) (Figure 2) capable of holding up to 25 kg of water was used. The internal structure allows water to move freely, transmitting inertial force and hydrodynamic instability to the wearer during exercise. Participants stood with their feet positioned at hip-width apart on marked locations on the floor, with their arms lightly crossed and fixed in front of the chest. They maintained a relaxed standing posture while looking straight ahead toward the camera, then performed the movement in synchronization with a metronome signal (60 bpm). Prior to the actual measurement, all participants received thorough instructions on the movement and practiced the lateral flexion movement several times at 60 bpm. When wearing the water vest, measurements were conducted immediately after confirming the movement tempo to prevent muscle fatigue. If errors in movement speed or execution occurred, the trial was temporarily suspended, and participants rested without the water vest while reconfirming the tempo. Participants performed lateral flexion to their comfortable end-range of motion for 4 complete cycles per side (8 cycles total). All trials were visually monitored by the same examiner to ensure consistency. Trials were repeated if excessive pelvic rotation, anterior–posterior trunk rotation, or changes in leg position were observed. A 3 min rest period was provided between load conditions, consistent with the American College of Sports Medicine recommendation of 2–5 min between sets for resistance exercise [17]. A progressive loading sequence (8 → 10 → 12 → 14 → 16 kg) was adopted, rather than randomization, to minimize injury risk, as sudden exposure to heavy unstable loads without prior lighter-load experience could compromise movement quality and participant safety. Although this design may introduce potential order effects such as fatigue or learning, the 3 min rest periods between conditions and the brief exercise duration (4 cycles per side per condition) were intended to minimize these effects. This limitation is further addressed in the Limitations Section.

## 3. Measurements

### 3.1. Surface Electromyography

**Figure 3:**
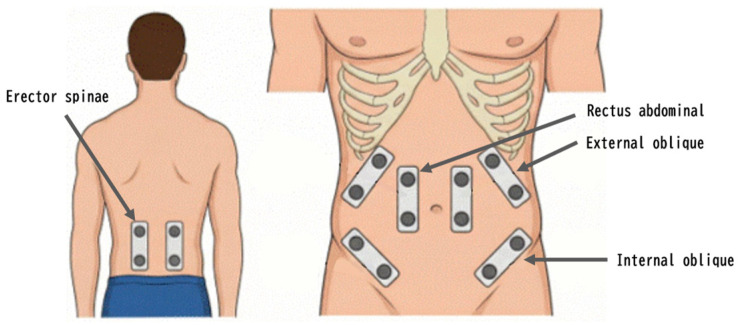
EMG electrode placement.

Surface EMG signals were recorded using Ultium EMG (Noraxon USA Inc., Scottsdale, AZ, USA) from four trunk muscles: rectus abdominis (RA), external oblique (EO), internal oblique (IO), and erector spinae (ES) (Figure 3). Bipolar Ag/AgCl electrodes were placed according to SENIAM [18] with 20 mm inter-electrode distance. Electrode sites were cleaned with alcohol and prepared with abrasive gel to minimize skin–electrode impedance. EMG signals were sampled at 1000 Hz and band-pass filtered (20–450 Hz). The filtered EMG signals were then full-wave rectified and smoothed using a root mean square (RMS) algorithm with a 50 ms moving window. The middle 2 cycles per side were selected for analysis to exclude the initial and final cycles, which may be affected by movement initiation and deceleration artifacts, respectively. Mean RMS amplitude of the selected cycles was calculated for each muscle and normalized to the respective MVIC value, expressed as %MVIC. Prior to MVIC testing, participants performed one submaximal practice contraction at approximately 50% effort for each muscle to familiarize themselves with the testing positions and contraction procedures. MVIC data for each muscle were obtained by performing maximal isometric contractions against manual resistance in specific positions known to elicit maximal activation: trunk flexion for rectus abdominis, lateral trunk flexion for external oblique, axial rotation for internal oblique, and prone trunk extension for erector spinae. For ES, MVIC was measured in a modified Biering-Sørensen position with the participant lying prone on a treatment table, the upper body extending beyond the table edge at the level of the anterior superior iliac spine, and the lower body secured by straps at the hips and ankles. The participant performed maximal trunk extension against manual resistance applied to the upper back [19,20]. All MVIC trials were performed by the same experienced examiner to minimize inter-operator variability. Each participant performed two maximal contractions of 3 s with a 60 s rest interval between trials, and the highest value was selected as the reference for normalization. This protocol is consistent with previously validated trunk muscle MVIC procedures [19,20], although the 3 s contraction during exercise was then normalized to each participant’s MVIC value and expressed as a percentage (%MVIC), allowing standardized comparison of muscle activation levels across participants and conditions. It should be acknowledged that some %MVIC values exceeded 100%, particularly for the external oblique and internal oblique muscles. This may be attributable to the shorter MVIC testing and dynamic exercise involving unpredictable internal forces. Dynamic exercises involving stretch–shortening cycles and reactive stabilization against unstable loads can elicit muscle activation above isometric MVIC reference values [21]. Vigotsky et al. [22] also noted that in dynamic exercises, the relationship between isometric MVIC and task EMG does not hold, as muscle length, contraction velocity, and neural drive differ substantially between conditions. Values exceeding 100% MVIC were therefore retained in the analysis, as they reflect the inherent limitation of isometric normalization rather than measurement error.

### 3.2. RPE Assessment

**Figure 4:**
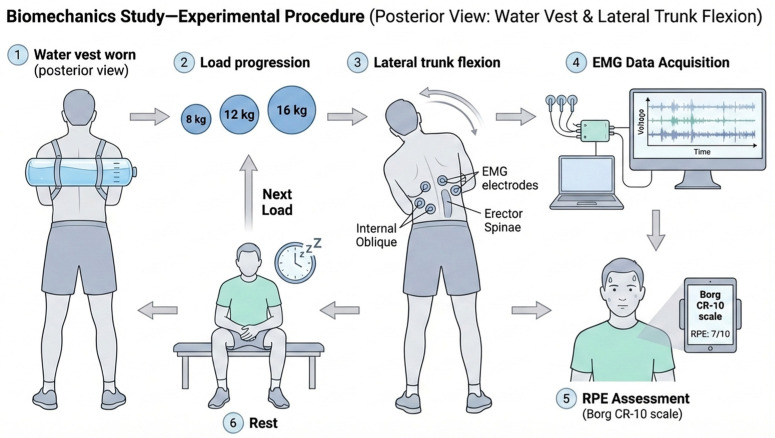
Schematic overview of the experimental procedure.

RPE was assessed using the Borg CR-10 scale immediately after completing each load condition. Participants rated their overall perceived exertion from 0 (no exertion at all) to 10 (maximal exertion). An overview of the measurement procedure is presented in (Figure 4).

## 4. Data Analysis

All data were analyzed using SPSS version 26.0 (SPSS Inc., Chicago, IL, USA). Descriptive statistics were presented as means and standard deviations. Shapiro–Wilk tests revealed that EO met the normality assumption (*p* = 0.344), whereas RPE, RA, IO, and ES violated normality (all *p* < 0.01). However, visual inspection of histograms and Q-Q plots indicated no severe departures from normality, and skewness and kurtosis values were within acceptable ranges (skewness: 0.175–1.440; kurtosis: −0.747–3.775) [23]. Given that repeated-measures ANOVA is robust to violations of normality, parametric statistics were applied.

One-way repeated-measures ANOVA was used to examine the effect of load on muscle activation. Mauchly’s test was used to assess sphericity, and Greenhouse–Geisser correction was applied when sphericity was violated. Effect sizes were calculated to assess relationships between RPE and muscle activation. Simple regression analyses (dependent variables: each muscle EMG and Total_Trunk; independent variables: Load and RPE) were conducted to develop prediction equations. Model fit was evaluated using the coefficient of determination (R^2^). Since the repeated-measures design resulted in non-independent observations nested within participants (N = 17, 85 observations), a linear mixed-effects model with a random intercept for participants was fitted to account for this hierarchical data structure. The fixed effect was RPE, and participants were modeled as a random effect (random intercept only, no random slopes) to account for individual baseline differences in trunk muscle activation. The simple regression model was prioritized for deriving the field-applicable prediction formula due to its practical simplicity, while the mixed-effects model served as a sensitivity analysis to verify that the fixed-effect estimates remained consistent after accounting for individual variability. For descriptive analysis of practical intensity guidelines, RPE scores were reclassified into three intensity categories based on Borg’s original CR-10 scale verbal anchors [11]: low (CR-10: 2–4, ‘weak’ to ‘somewhat strong’), moderate (CR-10: 5–6, ‘strong’), and high (CR-10: 7–10, ‘very strong’ to ‘extremely strong’).

## 5. Results

### 5.1. Changes in Trunk Muscle Activation with Increasing Load

Descriptive statistics for RPE and trunk muscle activation by load condition are presented in Table 2. Repeated-measures ANOVA was conducted to examine differences in muscle activation across water inertia load conditions (Table 3). Mauchly’s test of sphericity revealed violations for all muscles (RA: W = 0.049, *p* < 0.001; EO: W = 0.140, *p* = 0.001; IO: W = 0.063, *p* < 0.001; ES: W = 0.185, *p* = 0.004), and Greenhouse–Geisser corrected values were applied.

**Figure 5:**
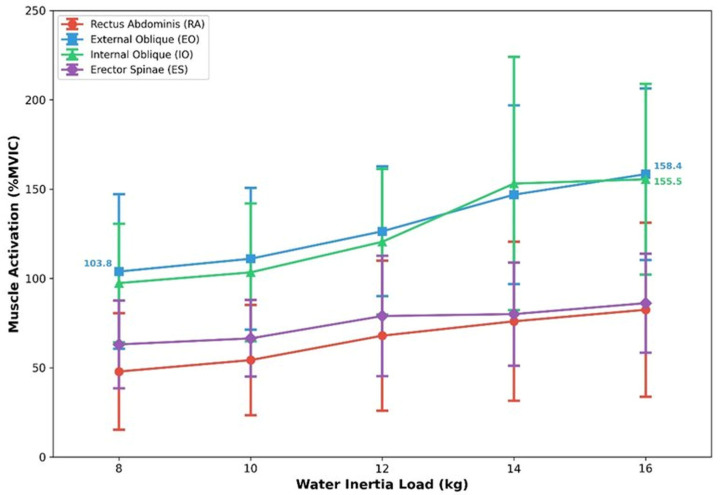
Trunk muscle activation (%MVIC) across water inertia loads (8–16 kg). Error bars represent ± 1 *SD*.

Analysis results showed significant differences in muscle activation according to water inertia load for all trunk muscles (all *p* < 0.001) (Figure 5). Percentage changes between 8 kg and 16 kg conditions were calculated as relative change [(16 kg value − 8 kg value)/8 kg value × 100]. EO showed the largest effect size (F (1.987, 31.785) = 18.209, *p* < 0.001, ηp^2^ = 0.532), increasing approximately 52% from 8 kg (103.8 ± 43.3%MVIC) to 16 kg (158.4 ± 48.1%MVIC). IO showed significant differences (F (1.756, 28.099) = 15.649, *p* < 0.001, ηp^2^ = 0.494), increasing by approximately 60% from 8 kg (97.4 ± 33.2% MVIC) to 16 kg (155.5 ± 53.4% MVIC). RA (F (1.799, 28.787) = 15.586, *p* < 0.001, ηp^2^ = 0.493) increased approximately 72% with the weight increase (47.8 ± 32.7%MVIC → 82.4 ± 48.7%MVIC). ES (F (2.304, 36.861) = 9.856, *p* < 0.001, ηp^2^ = 0.381) showed a relatively smaller effect size but still demonstrated a significant increase (63.0 ± 24.6%MVIC → 86.1 ± 27.7%MVIC, 37% increase). All ηp^2^ values represent large effect sizes according to Cohen’s [24] guidelines (large ≥ 0.14).

Bonferroni-corrected pairwise comparisons revealed distinct patterns of load sensitivity across muscles (Table 4). No muscle showed a significant difference between the 8 and 10 kg conditions. For RA, significant differences emerged between 8 kg and loads of 12 kg or higher (all *p* ≤ 0.005), and between 10 kg and loads of 14 kg or higher (all *p* ≤ 0.010), whereas adjacent conditions above 12 kg did not differ significantly. EO and IO demonstrated similar patterns, with significant differences generally requiring at least a 4–6 kg load increment. ES showed the least sensitivity to load changes, with significant differences observed only between 8 and 16 kg (*p* = 0.010) and between 10 kg and loads of 14 kg or higher (*p* ≤ 0.029). Across all muscles, adjacent load conditions (2 kg increments) rarely reached significance, suggesting that a minimum load difference of 4 kg is needed to produce statistically detectable changes in trunk muscle activation during water inertia load exercise.

### 5.2. Characteristics of Rating of Perceived Exertion

**Figure 6:**
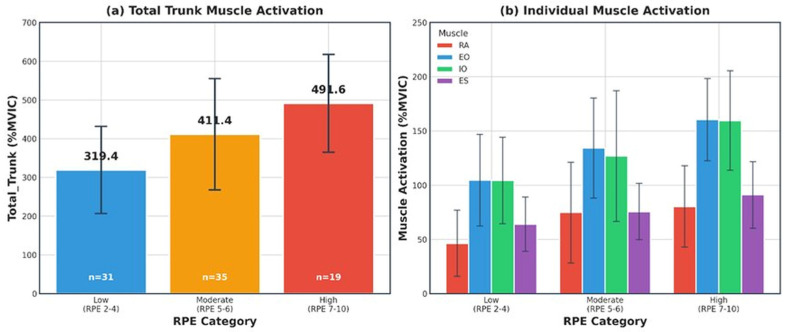
Trunk muscle activation across RPE categories.

RPE was measured using the Borg CR10 scale, with an overall mean of 5.2 ± 1.7 (range: 2–9). RPE showed a linear increase pattern with weight increase (8 kg: 3.4 ± 0.9; 10 kg: 4.2 ± 1.1; 12 kg: 5.2 ± 1.1; 14 kg: 6.2 ± 1.0; 16 kg: 7.0 ± 0.9), increasing approximately 0.9 points per weight increment. For practical application, RPE was reclassified into low intensity (CR10: 2–4), moderate intensity (CR10: 5–6), and high intensity (CR10: 7–10), resulting in relatively balanced distribution: low intensity, 31 trials (36.5%); moderate intensity, 35 trials (41.2%); and high intensity, 19 trials (22.4%) (Figure 6).

### 5.3. Correlation Between RPE and Muscle Activation

Pearson correlation analysis revealed significant positive correlations between RPE and all trunk muscle activation variables (Table 5). RPE showed the strongest correlation with EO (r = 0.46, *p* < 0.001), followed by IO (r = 0.43, *p* < 0.001), RA (r = 0.39, *p* < 0.001), and ES (r = 0.37, *p* < 0.001). Among the muscles, RA and EO demonstrated the highest inter-muscle correlation (r = 0.63, *p* < 0.001), while IO and ES showed the lowest (r = 0.37, *p* = 0.001). These results indicate that RPE reflects overall trunk muscle effort, with abdominal muscles showing stronger associations than the erector spinae. The inter-muscle correlation patterns also warrant discussion. The highest correlation between RA and EO (r = 0.63) likely reflects their synergistic role during lateral flexion, where both muscles contribute to trunk stabilization on the contralateral side. Conversely, the lowest correlation between IO and ES (r = 0.37) reflects their antagonistic functional relationship, with IO serving as a primary lateral flexor and ES primarily contributing to posterior stabilization against the anteriorly shifting water inertia load.

### 5.4. Simple Regression: Predictive Power of RPE for Individual Muscle Activation

Simple regression analysis was conducted to verify RPE’s predictive power for muscle activation (Table 6). Results showed that RPE significantly predicted activation of all trunk muscles (all *p* < 0.001). EO showed the highest explanatory power (R^2^ = 0.207, F (1, 83) = 21.648, *p* < 0.001, β = 0.455), with EO increasing by approximately 13.1%MVIC per 1-point increase in RPE. IO showed R^2^ = 0.186, RA showed R^2^ = 0.151, and ES showed R^2^ = 0.137, each demonstrating significant predictive power. This indicates that subjective RPE alone can predict approximately 14–21% of objective muscle activation.

### 5.5. Simple Regression Results for Composite Muscle Activation Indices

To control for individual differences in muscle activation strategies, additional analysis was conducted with a composite index summing the EMG of four muscles (Table 7). Total_Trunk EMG, the sum of %MVIC values for RA, EO, IO, and ES, represents total trunk muscle activation. Simple regression analysis showed that RPE significantly predicted Total_Trunk EMG (R^2^ = 0.267, F (1, 83) = 30.261, *p* < 0.001, β = 0.517), representing approximately 29% improvement over the highest individual muscle analysis (EO, R^2^ = 0.207). The prediction formula was ‘Total_Trunk EMG = 169.7 + 43.4 × RPE’, indicating that total muscle activation increases by approximately 43.4%MVIC per 1-point increase in RPE.

### 5.6. Multiple Regression: Combined Predictive Power of Load and RPE

Multiple regression analysis was conducted to examine changes in RPE’s predictive power when weight information was added (Table 8). For RA, the overall model was significant (R^2^ = 0.151, F (2, 82) = 7.305, *p* = 0.001), but only RPE emerged as a significant predictor (β = 0.385, *p* = 0.025), while LOAD was not significant (β = 0.004, *p* = 0.979). The same pattern was observed for ES. For EO and IO, adding LOAD weakened or rendered RPE non-significant. This is interpreted as the effect of multicollinearity due to high correlation between weight and RPE (r = 0.82), suggesting that adding weight information does not improve predictive power over RPE alone. Although the VIF value (2.74) was below the conventional multicollinearity threshold of 5–10 [25], the non-significance of LOAD likely reflects conceptual overlap rather than statistical multicollinearity. Because RPE inherently integrates the participant’s perception of load magnitude, the unique variance attributable to LOAD after accounting for RPE is minimal, resulting in a non-significant independent contribution.

### 5.7. Descriptive Statistics of Trunk Muscle Activation by RPE

To derive practical guidelines for field application, the mean and range of muscle activation by RPE category were analyzed (Table 9). Mean activation showed stepwise increases across all muscles as the RPE category increased. The composite trunk activation index increased approximately 54% from 319.4 points (mean 79.9%MVIC) at low intensity to 491.6 points (mean 122.9%MVIC) at high intensity.

### 5.8. Verification Through Multilevel Modeling

To account for the non-independence of observations due to the repeated-measure design, multilevel model analysis was additionally conducted. Results confirmed RPE as a significant predictor of Total_Trunk (Table 10), with significant random effects for participants (variance = 13,205.66, *p* = 0.006). The fixed-effect coefficient from the mixed-effects model (β = 46.08) was comparable to that obtained from the simple regression analysis (β = 43.4), indicating that the predictive relationship between RPE and Total_Trunk remained consistent after accounting for the hierarchical data structure. This consistency across modeling approaches supports the robustness of the simpler prediction formula for field application. A multilevel model was also fitted to account for the non-independence of repeated observations within participants.

## 6. Discussion

### 6.1. Summary of Main Results

This study aimed to verify whether RPE can predict trunk muscle activation during water inertia load trunk lateral flexion exercise and to develop a practical prediction model for field practitioners without EMG equipment. Seventeen healthy young adults (11 males, 6 females) were measured across five load levels (8–16 kg), yielding the following main results. First, trunk muscle activation significantly increased with load for all muscles, with large effect sizes (ηp^2^ = 0.381–0.532). Second, moderate positive correlations were found between RPE and individual trunk muscle activation (r = 0.37–0.46). Third, RPE explained 14–21% of variances in individual muscle activation. Fourth, the composite trunk activation index substantially improved prediction accuracy (R^2^ = 0.267), representing a 29% improvement over individual muscle analysis, and a field-applicable prediction formula (Total_Trunk = 169.7 + 43.4 × RPE) was derived.

### 6.2. Load-EMG Relationship: Comparison with Previous Studies

The significant increase in trunk muscle activation with water inertia load is consistent with previous research on unstable resistance training. Ditroilo et al. [4] reported increased activation of the external oblique and multifidus during isometric squats using water bags compared to traditional barbells. Lawrence and Carlson [26] also reported significantly increased activation of the rectus abdominis and external oblique during back squats using unstable loads, supporting that water inertia characteristics can induce similar muscle activation enhancement effects. In the present study, the external oblique (EO) showed the highest responsiveness to load increase (ηp^2^ = 0.532). This finding is consistent with the anatomical role of the EO as a primary mover in contralateral lateral flexion, where its oblique fiber orientation is optimally aligned to generate lateral flexion torque. However, this dominant EO responsiveness may be specific to the lateral flexion exercise used in the present study, as other movement patterns such as trunk flexion or rotation would likely emphasize different muscle groups, followed by the internal oblique (IO) (ηp^2^ = 0.494) and rectus abdominis (RA) (ηp^2^ = 0.493). The effects of training utilizing water inertia load have been verified across various populations. Kang and Park [6] reported that instability neuromuscular training using water inertia load significantly improved balance ability in healthy older women. Kang et al. [27] reported that dynamic neuromuscular stabilization training applying a water inertia load improved functional movement and postural sway in middle-aged women. Additionally, Kim and Park [10] confirmed that dynamic stability training using a water inertia load improved gait and biomechanical parameters in older women, and Huang et al. [7] reported positive effects of water inertia load training on lower extremity kinematics and center of pressure control during stair ambulation in middle-aged women with degenerative knee arthritis.

However, a water inertia load possesses fundamentally different characteristics from fixed loads such as dumbbells or kettlebells. While fixed loads have constant and predictable weight, water in water bags moves in a delayed manner opposite to the movement direction, generating unpredictable inertial forces [5]. This hydrodynamic instability means that even with the same absolute weight, the actually perceived load can vary depending on movement speed, direction change timing, and individual control ability. Therefore, in water inertia load training, exercise intensity cannot be simply defined by water weight (kg) alone, which is why subjective RPE becomes even more important as a complementary indicator for intensity prescription. Indeed, multiple regression analysis in this study showed no improvement in predictive power when the load variable was entered together with RPE, with only RPE confirmed as a significant predictor. This is interpreted as RPE already encompassing load information while also reflecting individual differences in perceived intensity due to water instability. Meanwhile, the non-significant predictive power of RPE for EO and IO can be attributed to these muscles being the primary movers in lateral flexion, showing the greatest sensitivity to load increases (ηp^2^ = 0.532 and 0.494, respectively). This likely resulted in greater shared variance between LOAD and RPE compared to other muscles. Notably, the EO showed marginally significant results (*p* = 0.067), suggesting potential significance with a larger sample size. Consequently, in training utilizing water inertia load, RPE has value not as a simple auxiliary indicator but as a core intensity indicator that can replace objective weight information.

### 6.3. RPE-EMG Correlation: Comparison with Previous Studies

The observed moderate correlations between RPE and trunk muscle activation (r = 0.37–0.46) need to be interpreted in comparison with previous studies validating RPE in resistance exercise. Lea et al. [12] reported an integrated validity coefficient of r = 0.88 between RPE and various physiological indicators during resistance exercise through meta-analysis. However, sub-analysis targeting only EMG in that study showed a wide range of correlation coefficients, reflecting variability according to exercise type and measured muscles. The relatively lower correlation coefficients in this study compared to previous research appear to be attributable to the unique characteristics of water inertia load exercise. First, water inertia load provides continuously varying resistance throughout movement, resulting in greater variability in muscle activation patterns compared to traditional resistance exercises using fixed loads. Second, individual differences in stabilization strategies to control unstable loads may have weakened the RPE-EMG relationship. Lagally et al. [28] reported that RPE shows significant correlations with EMG and blood lactate during resistance exercise, but the strength varies according to exercise modality and measured muscle groups. Helms et al. [15] reported that RPE-based load prescription induced similar or better strength improvements than %1RM-based prescription, providing additional support for the validity of RPE as a training intensity indicator. It should also be considered that the moderate RPE-EMG correlations observed in this study may partly reflect the unique perceptual demands of water inertia load exercise. Unlike fixed-weight resistance training, where perceived exertion closely corresponds to actual muscular effort, participants exercising with water inertia load may perceive the exercise as more demanding than the objective muscle activation levels indicate. This discrepancy likely arises from the central nervous system’s continuous effort to anticipate and counteract the irregular, time-delayed fluid movements within the vest, generating additional neural drive for reactive trunk stabilization that elevates perceived exertion beyond what peripheral muscle activation alone would predict. Despite this inherent complexity, the correlation coefficients of r = 0.37–0.46 (all *p* < 0.001) remain within a clinically meaningful range for field application, as moderate correlations (r = 0.30–0.50) can still provide useful guidance for exercise prescription, particularly when combined with composite indices that substantially improve prediction accuracy. Future research with larger sample sizes and a broader range of trunk muscles and movement patterns will be essential to further elucidate the RPE-EMG relationship under unstable loading conditions and to expand the practical applicability of RPE-based intensity prescription in water inertia load training.

### 6.4. Limitations of Individual Muscle Prediction

Simple regression analysis showed that RPE explained 14–21% of the variance in individual trunk muscle activation. According to Cohen’s [24] criteria, this corresponds to a medium effect size (R^2^ = 0.13–0.26). However, these values are notably lower than the R^2^ = 0.50–0.85 reported by Cruz-Montecinos et al. [29] between RPE and neuromuscular fatigue indicators of trunk muscles during a plank exercise. This difference can be explained by several factors. First, Cruz-Montecinos et al.’s [29] study used isometric planks, minimizing movement variability, whereas the lateral flexion exercise in this study has dynamic characteristics resulting in greater variation in muscle activation patterns. Second, the unstable characteristics of water inertia load may have increased inter-individual variability by causing participants to adopt different muscle recruitment strategies. Third, Cruz-Montecinos et al. [29] performed exercise until exhaustion to induce fatigue, whereas this study measured only four cycles at each load condition, limiting fatigue accumulation. It is important to note that the R^2^ values (0.50–0.85) reported by Cruz-Montecinos et al. [29] were obtained for predicting neuromuscular-fatigue indicators (e.g., median frequency decline) rather than muscle activation amplitude per se. Fatigue-related EMG changes during sustained isometric exercise follow a more predictable trajectory than dynamic muscle activation patterns, which may account for the higher predictive accuracy observed in their study. Moreover, a review of the existing literature reveals that most previous studies have examined the RPE-EMG relationship only in terms of correlation coefficients during conventional resistance exercises using fixed loads, and no prior study has attempted to develop an RPE-based prediction model for trunk muscle activation during exercise involving unpredictable instability such as water inertia load. The inherently variable and reactive nature of water inertia load exercise presents unique challenges for prediction modeling that do not exist in traditional stable or even predictable unstable conditions. Therefore, despite the moderate R^2^ values observed in the present study, the attempt to establish a quantitative prediction framework in this novel exercise modality represents a meaningful contribution to the field and provides a foundation upon which future studies can build with larger samples, diverse muscle groups, and varied movement patterns.

### 6.5. Superiority of Composite Index: Core Contribution of This Study

The core contribution of this study is the development and validation of the composite trunk activation index. The Total_Trunk index, summing normalized EMG values of four trunk muscles (RA, EO, IO, ES), achieved R^2^ = 0.267, representing a 29% improvement over the highest individual muscle predictor (EO, R^2^ = 0.207). Batista et al. [3] also confirmed that unstable conditions significantly increase activation of all individual core muscles, supporting the validity of the composite index approach. According to Cohen’s [24] criteria, individual muscles remained at medium effect size (R^2^ = 0.13–0.26), whereas the composite index exceeded the large-effect-size criterion (R^2^ > 0.26). This improvement is attributable to the statistical principle that combining multiple measurements reduces random error while preserving the systematic signal. Previous studies mainly examined RPE-EMG relationships for individual muscles, but no research had proposed an integrated index for predicting total trunk muscle load during unstable resistance exercise. The prediction formula (Total_Trunk = 169.7 + 43.4 × RPE) provides practitioners with a simple tool to estimate total trunk muscle activation using only subjective exertion assessment. While R^2^ = 0.267 indicates that approximately 73% of the variance in trunk muscle activation remains unexplained, this level of prediction accuracy should be interpreted within the context of field application. In settings where no EMG equipment is available, even moderate predictive accuracy provides practitioners with a useful screening-level tool for estimating exercise intensity. The prediction formula is not intended to replace EMG assessment but rather to offer an accessible alternative for general intensity prescription in water inertia load training. Nevertheless, the unexplained variance highlights the need for future studies with larger sample sizes to develop more robust prediction models. Additionally, incorporating a broader range of trunk muscles beyond the four examined in this study, as well as testing across different movement patterns (flexion, extension, rotation), may capture additional variance and improve prediction accuracy. Until such studies are conducted, the current formula should be applied conservatively as a preliminary reference rather than a definitive prescription tool.

### 6.6. Practical Applications

RPE category-based trunk muscle activation classification provides practical guidelines for exercise prescription. Low-intensity exercise (RPE 2–4) induced approximately 79.9%MVIC mean trunk muscle activation, moderate intensity (RPE 5–6) showed 102.9%MVIC, and high intensity (RPE 7–10) showed 122.9%MVIC mean per muscle. In dynamic exercise, muscle activation exceeding isometric MVIC can occur due to stretch reflex and instability compensation [21]. These reference values enable practitioners to prescribe water inertia load training intensity without EMG equipment. This approach aligns with autoregulation principles emphasized in modern resistance training. Helms et al. [30] proposed that RPE-based intensity prescription can automatically adjust training load to reflect individual daily condition variations, and Greig et al. [31] confirmed that RPE-based autoregulation is a valid method for training load monitoring.

### 6.7. Limitations

This study has several limitations. First, the sample consisted of healthy young adults with regular exercise experience (11 males, 6 females), potentially limiting generalization to other populations such as the elderly or clinical groups. Although females were included in this study, subgroup analysis by sex was not performed due to the limited sample size. Second, the repeated-measures design with the same participants (N = 17, 85 observations) may raise concerns about the non-independence of observations. To address this, multilevel model analysis was additionally conducted. Results showed that despite significant inter-individual variability (variance = 13,205.66, *p* = 0.006), the predictive power of RPE remained significant (β = 46.08, *p* < 0.001). This suggests that the prediction model in this study is statistically robust despite the limited sample size. However, future studies should recruit larger samples to develop more sophisticated prediction models based on multilevel modeling. Third, since only lateral flexion movement was examined, prediction relationships may differ in other trunk movements such as flexion, extension, and rotation. Fourth, the moderate R^2^ value (composite index 0.267) indicates that a substantial portion of trunk muscle activation is not explained by RPE alone. Fifth, the prediction formula derived in this study (Total_Trunk = 169.7 + 43.4 × RPE) provides practitioners with a simple tool to estimate total trunk muscle activation using only subjective exertion assessment. It should be noted that this formula is intended for practitioners to understand what level of trunk muscle activation corresponds to a given RPE value, rather than for real-time EMG calculation in the field. For example, if a practitioner aims to prescribe moderate-intensity trunk activation (approximately 100%MVIC per muscle, or Total_Trunk ≈ 400%MVIC), the corresponding target RPE would be approximately 5.3 on the CR-10 scale [(400 − 169.7)/43.4 ≈ 5.3], indicating that participants should maintain a perceived exertion of ‘strong’ during the exercise. However, as this formula was developed within the same sample, and cross-validation using an independent sample was not performed, the prediction accuracy should be interpreted with caution. Due to the preliminary nature of this study, the sample size (N = 17) was insufficient for training–validation set splitting. Therefore, external validation in an independent sample is needed to confirm the generalizability of this formula. Sixth, some muscles exhibited %MVIC values exceeding 100%, particularly the external oblique and internal oblique. This is a recognized limitation of isometric MVIC normalization during dynamic tasks [21]. The unpredictable inertial forces generated by water movement likely elicited reactive neuromuscular responses that exceeded the activation levels achievable during controlled isometric contractions. While this does not invalidate the relative comparisons across load conditions within this study, it should be considered when interpreting absolute %MVIC values. Seventh, the MVIC contraction duration of 3 s was shorter than the 5 s recommended by Konrad [32], which may have contributed to submaximal reference values and consequently inflated %MVIC during dynamic exercise. However, it should also be noted that prolonged maximal isometric contractions sustained beyond a few seconds can induce rapid fatigue, potentially compromising the reliability of the MVIC reference value [33]. Balancing these considerations, a 3 s protocol was adopted following previously validated trunk muscle MVIC procedures [19,20]. Nonetheless, future studies should consider adopting longer contraction durations or multiple MVIC positions to ensure more robust normalization reference values. Eighth, the progressive (non-randomized) load sequence may have introduced order effects, including cumulative fatigue and motor learning across successive conditions. Although 3 min rest periods were provided, the possibility that later load conditions were influenced by prior exercise cannot be excluded. Ninth, the lack of cross-validation in an independent sample is a critical limitation. The prediction equation should be considered preliminary until validated in a separate cohort with different characteristics. Tenth, the relatively short exercise duration (four cycles per side per condition) may not represent typical training sets, and the RPE-EMG relationship during prolonged exercise with greater fatigue accumulation may differ from the present findings. Eleventh, results are specific to the lateral flexion movement pattern and the particular water vest design used (SMARTFIT AQUA VEST); generalizability to different movement patterns (flexion, extension, rotation) and other water inertia load equipment requires further investigation.

## 7. Conclusions

This study demonstrated that RPE can significantly predict trunk muscle activation during water inertia load trunk lateral flexion exercise. While individual muscle analysis showed only 14–21% explanatory power, the composite trunk activation index, summing four muscles, achieved 26.7% explanatory power, representing a 29% improvement. This suggests that composite-index approaches are effective for controlling individual differences in muscle activation strategies under unstable load environments.

The derived prediction formula (Total_Trunk = 169.7 + 43.4 × RPE) and RPE category-based muscle activation reference values (low intensity 79.9%MVIC, moderate intensity 102.9%MVIC, high intensity 122.9%MVIC) are provided as practical tools enabling field practitioners to prescribe water inertia load training intensity without EMG equipment. In training environments where intensity cannot be simply defined by objective weight, RPE has value as a core intensity indicator. These findings should be considered preliminary, given the pilot study design and small sample size, and the prediction formula requires cross-validation in an independent sample before widespread adoption. Future research should verify these findings across diverse populations (the elderly and clinical groups) and movement patterns (flexion, extension, rotation), and develop more sophisticated prediction models that appropriately account for individual variation through multilevel modeling analysis.

## Figures and Tables

**Table 1:** Characteristics of study participants (N = 17).

	Height (cm)	Weight (kg)	Age (yrs)	BMI (kg/m^2^)
M (*n* = 11)	174.09 ± 6.22	70.55 ± 8.98	20.45 ± 2.02	23.19 ± 1.66
F (*n* = 6)	162.67 ± 5.01	59.83 ± 9.93	19.67 ± 0.52	22.51 ± 2.65

*Note*. Values are presented as means ± standard deviations. M = male; F = female; BMI = body mass index.

**Table 2:** Descriptive statistics of RPE and trunk muscle activation by load condition.

Load (kg)	RPE	RA	EO	IO	ES	Total Trunk
8	3.41 ± 0.94	47.83 ± 32.66	103.84 ± 43.32	97.37 ± 33.24	63.02 ± 24.58	312.06 ± 104.13
10	4.18 ± 1.13	54.23 ± 30.99	111.00 ± 39.71	103.36 ± 38.65	66.39 ± 21.46	334.98 ± 109.34
12	5.24 ± 1.09	67.91 ± 42.00	126.36 ± 36.30	120.49 ± 40.69	78.92 ± 33.67	393.69 ± 123.37
14	6.24 ± 0.97	75.96 ± 44.57	146.87 ± 50.05	153.09 ± 70.94	80.00 ± 28.89	455.92 ± 138.87
16	7.00 ± 0.94	82.43 ± 48.73	158.36 ± 48.05	155.50 ± 53.43	86.13 ± 27.72	482.42 ± 141.41

*Note*. *N* = 17 per load condition. Values are presented as *M* ± *SD*. Muscle activation expressed as %MVIC; RPE measured using Borg CR-10 scale. RA = rectus abdominis; EO = external oblique; IO = internal oblique; ES = erector spinae; Total Trunk = sum of %MVIC for RA, EO, IO and ES.

**Table 3:** Trunk muscle activation across increasing loads.

Muscle	F	df (GG)	*p*	ηp^2^
RA	15.586	1.799, 28.787	<0.001	0.493
EO	18.209	1.987, 31.785	<0.001	0.532
IO	15.649	1.756, 28.099	<0.001	0.494
ES	9.856	2.304, 36.861	<0.001	0.381

*Note*. *N* = 17. GG = Greenhouse–Geisser correction; ηp^2^ = partial eta squared. Greenhouse–Geisser-corrected values were applied due to violations of Mauchly’s test of sphericity across all muscles. RA = rectus abdominis; EO = external oblique; IO = internal oblique; ES = erector spinae.

**Table 4:** Bonferroni-corrected pairwise comparisons of trunk muscle activation between load conditions.

Comparison (kg)	RA MD (*p*)	EO MD (*p*)	IO MD (*p*)	ES MD (*p*)
8 vs. 10	−6.4 (0.060)	−7.2 (1.000)	−6.0 (1.000)	−3.4 (1.000)
8 vs. 12	−20.1 (0.005 *)	−22.5 (0.172)	−23.1 (0.003 *)	−15.9 (0.117)
8 vs. 14	−28.1 (0.003 *)	−43.0 (0.009 *)	−55.7 (0.018 *)	−17.0 (0.082)
8 vs. 16	−34.6 (0.001 *)	−54.5 (0.002 *)	−58.1 (<0.001 *)	−23.1 (0.010 *)
10 vs. 12	−13.7 (0.058)	−15.4 (0.022 *)	−17.1 (0.147)	−12.5 (0.066)
10 vs. 14	−21.7 (0.010 *)	−35.9 (0.001 *)	−49.7 (0.019 *)	−13.6 (0.029 *)
10 vs. 16	−28.2 (0.008 *)	−47.4 (<0.001 *)	−52.1 (<0.001 *)	−19.7 (0.001 *)
12 vs. 14	−8.0 (0.868)	−20.5 (0.053)	−32.6 (0.135)	−1.1 (1.000)
12 vs. 16	−14.5 (0.262)	−32.0 (0.001 *)	−35.0 (0.004 *)	−7.2 (1.000)
14 vs. 16	−6.5 (0.433)	−11.5 (0.439)	−2.4 (1.000)	−6.1 (0.189)

*Note*. MD = mean difference (earlier load − later load); values in parentheses are Bonferroni-adjusted *p*-values. RA = rectus abdominis; EO = external oblique; IO = internal oblique; ES = erector spinae. * *p* < 0.05.

**Table 5:** Correlation coefficients among RPE and muscle activation variables.

Variable	1	2	3	4	5
RPE	—				
RA	0.39 **	—			
EO	0.46 **	0.63 **	—		
IO	0.43 **	0.59 **	0.52 **	—	
ES	0.37 **	0.51 **	0.51 **	0.37 **	—

*Note*. *N* = 85. RPE = rating of perceived exertion; RA = rectus abdominis; EO = external oblique; IO = internal oblique; ES = erector spinae. ** *p* < 0.01.

**Table 6:** Simple regression result: Predictive power of RPE for individual muscle activation.

Muscle	R^2^	F	df	*p*	β	B	SE	95% CI	Intercept	Prediction Formula
RA	0.151	14.788	1, 83	<0.001	0.389	9.794	2.547	[4.73, 14.86]	14.630	RA = 14.6 + 9.8 × RPE
EO	0.207	21.648	1, 83	<0.001	0.455	13.118	2.819	[7.51, 18.73]	60.918	EO = 60.9 + 13.1 × RPE
IO	0.186	18.940	1, 83	<0.001	0.431	14.115	3.243	[7.66, 20.57]	52.397	IO = 52.4 + 14.1 × RPE
ES	0.137	13.219	1, 83	<0.001	0.371	6.366	1.751	[2.88, 9.85]	41.714	ES = 41.7 + 6.4 × RPE

*Note*. Dependent variable: EMG (%MVIC) for each muscle. Independent variable: RPE. SE = b standard error of B. 95% CI = 95% confidence interval for B. RA = rectus abdominis; EO = external oblique; IO = internal oblique; ES = erector spinae.

**Table 7:** Simple regression results for composite muscle activation indices.

Index	Components	R^2^	ΔR^2^ *	F	df	*p*	β	B	SE	95% CI	Prediction Formula
Total_ABD	RA + EO + IO	0.254	+23%	28.279	1, 83	<0.001	0.504	37.027	6.963	[23.18, 50.88]	127.9 + 37.0 × RPE
Total_Trunk	RA + EO + IO + ES	0.267	+29%	30.261	1, 83	<0.001	0.517	43.393	7.888	[27.71, 59.08]	169.7 + 43.4 × RPE

*Note*. Independent variable: RPE. Total_ABD = sum of three abdominal muscles; Total_Trunk = sum of all four muscles (unit: sum of %MVIC). 95% CI = 95% confidence interval for B. * ΔR^2^ = improvement rate compared to the highest individual muscle value (EO, R^2^ = 0.207).

**Table 8:** Multiple regression results: Load and RPE predicting trunk muscle activation.

DV	R^2^	F (2, 82)	*p*	β LOAD	*p*	β RPE	*p*	VIF
RA	0.151	7.305	0.001	0.004	0.979	0.385	0.025 *	2.74
EO	0.221	11.614	<0.001	0.195	0.231	0.299	0.067	2.74
IO	0.210	10.899	<0.001	0.258	0.117	0.226	0.169	2.74
ES	0.137	6.534	0.002	0.014	0.933	0.359	0.037 *	2.74
TotalTrunk	0.278	15.785	<0.001	0.172	0.272	0.380	0.017 *	2.74

*Note*. *N* = 85. DV = dependent variable; β = standardized coefficient; VIF = variance inflation factor. RA = rectus abdominis; EO = external oblique; IO = internal oblique; ES = erector spinae; Total Trunk = sum of %MVIC for four muscles. * *p* < 0.05.

**Table 9:** Descriptive statistics of trunk muscle activation by RPE category.

RPE Category	*N*	RA *M* (*SD*)	EO *M* (*SD*)	IO *M* (*SD*)	ES *M* (*SD*)	Total_Trunk (*SD*)	Mean *
Low (2–4)	31	46.4 (30.4)	104.6 (42.3)	104.4 (39.9)	64.0 (25.0)	319.4 (112.5)	79.9
Moderate (5–6)	35	74.7 (46.5)	134.2 (46.0)	126.8 (60.1)	75.7 (25.9)	411.4 (143.8)	102.9
High (7–10)	19	80.4 (37.3)	160.5 (38.0)	159.6 (45.8)	91.1 (30.7)	491.6 (126.4)	122.9

*Note*. *M* (*SD*) = mean (standard deviation), unit: %MVIC. Total_Trunk = sum of %MVIC for four muscles. * Mean = Total_Trunk ÷ 4, representing average activation across four muscles.

**Table 10:** Multilevel model results for predicting Total_Trunk from RPE. (**a**) Fixed effects, (**b**) random effects.

(a)					
Parameter	β	SE	df	*t*	*p*
Intercept	151.90	33.84	22.45	4.49	<0.001
RPE	46.08	3.73	58.75	12.35	<0.001
(**b**)					
**Parameter**	***Variance***		***SD***		***p***
Intercept [Subject]	13,205.66		114.92		0.006
Residual	5765.38		75.93		-

*Note*. *N* = 85 observations from 17 participants. β = unstandardized coefficient; SE = standard error; *SD* = standard deviation.

## Data Availability

The data used and/or analyzed during the current study are available from the corresponding author upon reasonable request.

## References

[1] Behm D.G., Anderson K.G. (2006). The role of instability with resistance training. J. Strength Cond. Res..

[2] Oliva-Lozano J.M., Muyor J.M. (2020). Core muscle activity during physical fitness exercises: A systematic review. Int. J. Environ. Res Public Health.

[3] Batista G.D.A., Beltrán S.P., Pereira dos Passos M.H., Calixtre L.B., Santos L.R.D.H., Cappato de Araújo R. (2024). Comparison of the electromyography activity during exercise with stable and unstable surfaces: A systematic review and meta-analysis. Sports.

[4] Ditroilo M., O’Sullivan R., Harnan B., Crossey A., Gillmor B., Dardis W., Grainger A. (2018). Water-filled training tubes increase core muscle activation and somatosensory control of balance during squat. J. Sports Sci..

[5] Glass S.C., Blanchette T.W., Karwan L.A., Pearson S.S., O’Neil A.P., Karlik D.A. (2016). Core muscle activation during unstable bicep curl using water-filled instability training tube. J. Strength Cond. Res..

[6] Kang S., Park I. (2024). Effects of instability neuromuscular training using an inertial load of water on the balance ability of healthy older women: A randomized clinical trial. J. Funct. Morphol. Kinesiol..

[7] Huang Y., Kang S., Park I. (2025). Effects of unstable exercise using the inertial load water on lower extremity kinematics and center of during stair ambulation in middle-aged women with degenerative knee arthritis. Appl. Sci..

[8] Calatayud J., Borreani S., Martin J., Martin F., Flandez J., Colado J.C. (2015). Core muscle activity in a series of balance exercises with different stability conditions. Gait Posture.

[9] Wezenbeek E., Verhaeghe L., Laveyne K., Ravelingien L., Witvrouw E., Schuermans J. (2022). The effect of aquabag use on muscle activation in functional strength training. J. Sport. Rehabil..

[10] Kim H.J., Park I.B. (2025). Effects of dynamic stability training with water inertia load on gait and biomechanics in older women: A randomized clinical trial. J. Funct. Morphol. Kinesiol..

[11] Borg G.A. (1982). Psychophysical bases of perceived exertion. Med. Sci. Sports Exerc..

[12] Lea J.W., O’Driscoll J.M., Hulbert S., Scales J., Wiles J.D. (2022). Convergent validity of ratings of perceived exertion during resistance exercise in healthy participants: A systematic review and meta-analysis. Sports Med. Open..

[13] Fontes E.B., Smirmaul B.P.C., Nakamura F.Y., Pereira G., Okano A.H., Altimari L.R., Dantas J.L., de Moraes A.C. (2010). The relationship between rating of perceived exertion and muscle activity during exhaustive constant-load cycling. Int. J. Sports Med..

[14] Lagally K.M., McCaw S.T., Young G.T., Medema H.C., Thomas D.Q. (2004). Rating of perceived exertion and muscle activity during the bench press exercise in recreational and novice lifters. J. Strength Cond. Res..

[15] Helms E.R., Byrnes R.K., Cooke D.M., Haischer M.H., Carzoli J.P., Johnson T.K., Cross M.R., Cronin J.B., Storey A.G., Zourdos M.C. (2018). RPE vs. percentage 1RM loading in periodized programs matched for sets and repetitions. Front. Physiol..

[16] Zhao H., Seo D., Okada J. (2023). Validity of using perceived exertion to assess muscle fatigue during back squat exercise. BMC. Sports Sci. Med. Rehabil..

[17] American College of Sports Medicine (2009). Progression Models in Resistance Training for Healthy Adults. Med. Sci. Sports Exerc..

[18] Stegeman D.F., Hermens H.J. (2007). Standard for surface electromyography: The European project Surface EMG for non-invasive assessment of muscles (SENIAM). Enschede Roessingh Res. Dev..

[19] Ng J.K.F., Keppers V., Parnianpour M., Richardson C.A. (2002). EMG activity normalization for trunk muscles in subjects with and without back pain. Med. Sci. Sports Exerc..

[20] Vera-Garcia F.J., Moreside J.M., Mcgill S.M. (2010). MVC techniques to normalize trunk muscle EMG in healthy women. J. Electromyogr. Kinesiol..

[21] Burden A. (2010). How should we normalize electromyograms obtained from healthy participants? What we have learned from over 25 years of research. J. Electromyogr. Kinesiol..

[22] Vigotsky A.D., Halperin I., Lehman G.J., Trajano G.S., Vieira T.M. (2018). Interpreting signal amplitudes in surface electromyography studies in sports and rehabilitation sciences. Front Physiol..

[23] Kline R.B. (2023). Principles and Practice of Structural Equation Modeling.

[24] Cohen J. (1988). Statistical Power Analysis for the Behavioral Sciences.

[25] Hair J.F., Black W.C., Babin B.J., Anderson R.E. (2019). Multivariate Data Analysis.

[26] Lawrence M.A., Carlson L.A. (2015). Effects of an unstable load on force and muscle activation during a parallel back squat. J. Strength Cond. Res..

[27] Kang S., Park I., Ha M.S. (2024). Effect of dynamic neuromuscular stabilization training using the inertial load of water on functional movement and postural sway in middle-aged women: A randomized controlled trial. BMC Womens Health.

[28] Lagally K.M., Robertson R.J., Gallagher K.I., Goss F.L., Jakicic J.M., Lephart S.M., McCaw S.T., Goodpaster B. (2002). Perceived exertion, electromyography, and blood lactate during acute bouts of resistance exercise. Med. Sci. Sports Exerc..

[29] Cruz-Montecinos C., Bustamante A., Candia-González M., González-Bravo C., Gallardo-Molina P., Andersen L., Calatayud J. (2019). Perceived physical exertion is a good indicator of neuromuscular fatigue for the core muscles. J. Electromyogr. Kinessiol..

[30] Helms E.R., Kwan K., Sousa C.A., Cronin J.B., Storey A.G., Zourdos M.C. (2020). Methods for regulating and monitoring resistance training. J. Hum. Kinet..

[31] Greig L., Hemingway B.H., Aspe R.R., Cooper K., Comfort P., Swinton P.A. (2020). Autoregulation in resistance training: Addressing the inconsistencies. Sports Med..

[32] Konrad P. (2005). The ABC of EMG: A Practical Introduction to Kinesiological Electromyography.

[33] Halaki M., Ginn K.A., Naik G.R. (2012). Normalization of EMG signals: To normalize or not to normalize and what to normalize to?. Computational Intelligence in Electromyography Analysis—A Perspective on Current Applications and Future Challenges.

